# Sexual Dysfunction in Carcinoma Cervix: Assessment in Post Treated Cases by LENTSOMA Scale

**DOI:** 10.31557/APJCP.2020.21.2.349

**Published:** 2020

**Authors:** Abhishek Shankar, Jaineet Patil, Anil Luther, Kavita Mandrelle, Abhijit Chakraborty, Anusha Dubey, Deepak Saini, Ram Pukar Bharat, Deepak Abrol, Sachidanand Jee Bharati, Veronika Bentard

**Affiliations:** 1 *Preventive Oncology, Dr BR Ambedkar Institute Rotary Cancer Hospital, All India Institute of Medical Sciences, Delhi, *; 2 *Radiation Oncology, *; 3 *General Surgery, *; 4 *Obstetrics & Gynecology, Christian Medical College, Ludhiana, *; 6 *Indian Society of Clinical Oncology, *; 7 *Radiation Oncology,*; 9 *Oncoanaesthesia and Palliative Medicine, Dr BR Ambedkar Institute Rotary Cancer Hospital, All India Institute of Medical Sciences, Delhi, *; 8 *Radiation Oncology, Government Medical College, Kathua, JK, India, *; 5 *The Baylor College of Medicine, Houston, TX, USA, *; 10 *Department of Tumour Biochemistry, Oncopharmacology, RE Kavetsky Institute of Experimental Pathology, Oncology and Radiobiology, NASU, Kiev, Ukraine. *

**Keywords:** Cervical Cancer- sexual dysfunction- LENTSOMA scales

## Abstract

Treatment for cervical cancer consists of hysterectomy, radiotherapy, chemotherapy and targeted therapy in different combination based on stage at presentation. However, late consequences of such radical treatments are known but not many Indian studies have reported it. Quality of life and impact on sexual health has become an important issue in view of long survival of cervical cancer patients. LENTSOMA scale is one such scale validated for scoring radiotherapy related morbidity. However, there is need for a comprehensive scale covering all aspects of physical and psychological disruptions to provide complete recovery and rehabilitation. The study was prospective and patients who were treated for cervical cancer on follow up were included in this study. A total of 85 patients, who were treated with surgery, radiotherapy, chemotherapy alone or in combination, comprising of stage I to stage IV disease, participated in this study. Findings of this study showed that pain during intercourse and altered sexual life were reported by 32.9% and 25.9% patients respectively whereas 24.7% found it problematic and in 22.3% patients, alteration in interest in sex were reported. Vaginal stenosis was seen in 75.29% of patients after treatment with decreased frequency of intercourse after treatment was seen in 16.4 % of patients. Combination of surgery and radiotherapy in cervical cancer patients caused more sexual dysfunction and dissatisfaction, especially in lower age group. Treatment morbidity in term of sexual function was more with advanced stage disease and with the patients on longer follow up. Sexual function is an important aspect of quality of life but there is no single self-report measure in routine clinical follow up use which is brief, easy to complete and incorporates all (physical, psychological, emotional) aspects of sexual health for people affected by cancer.

## Introduction

Globally 27% of total cervical cancer cases are from India which is home to 16-17% of world’s women population (Roy et al., 2018). Over the past 40 years mortality from carcinoma of the cervix has fallen due to improved treatment and the introduction of national screening programs (Shankar et al., 2018). Treatment for cervical cancer consists of hysterectomy, radiotherapy, chemotherapy and targeted therapy in different combination based on stage at presentation. Advances in treatment modalities and introduction of screening programs have resulted in improved survival rates. However, late consequences of such radical treatments are both under recognized as well as under reported (Davidson and Faithfull, 2006; Andreyev et al., 2010). Most of the diagnosed women belong to the sexually active age group with mean age at diagnosis approximately 50 years (Vistad et al., 2006). Therefore quality of life and impact on sexual health has become an important issue. Published studies suggest that cervical cancer survivors experience sexual difficulties after pelvic radiotherapy in 30–63% cases (Sadovsky et al., 2010; Jephcott et al., 2004; Jensen and Froeding, 2015). 

Surgery, radiotherapy and chemotherapy all have an impact on sexual health in several ways (Shankar et al., 2017). Sexual dysfunction after radiotherapy consists of physical and psychological impacts. Physical effects include disorders of arousal, orgasm and dyspareunia (Paul et al., 2005). Generally, pain during intercourse is found to be more common than arousal difficulties. Pain during intercourse after cervical cancer treatment is mainly a result of vaginal dryness, stenosis and shortening. Radical surgery leads to adhesion formation, in turn, leading to vaginal shortening (Hsu et al., 2009). Radiotherapy more commonly leads to stenosis and fibrosis along with loss of vaginal lubrication (Abitbol and Davenport, 1974). Some patients also present with vaginal bleeding. Psychological impacts include anxiety, depression, fear of altered body image and decreased sexual satisfaction in both patient and partner (White et al., 2013). As a result, survivors suffer from distressed marital relations and personal difficulties (Andersen, 1993). 

For patients with cervical cancer, the objective of new treatment approaches is to improve their survival without compromising Quality of Life (Shankar et al., 2019). In order to ensure holistic cancer care for survivors, it is important to incorporate both assessment and management of late treatment effects into routine clinical follow up. There are some existing scales based on patient reported outcomes for sexual dysfunction specifically like Female Sexual Function Index (FSFI) (Rosen et al., 2000) as well as for Quality of Life as a whole (Vistad et al., 2006; Ye Et al., 2014). LENTSOMA is one such scale validated for scoring radiotherapy related morbidity (Grigsby et al., 1995). However, there is need for a comprehensive scale covering all aspects of physical and psychological disruptions to provide complete recovery and rehabilitation.

## Materials and Methods

The study was prospective and patients who were treated for cervical cancer attending radiation oncology outpatient department for follow up were included in this study. A total of 85 patients, who were treated with surgery, radiotherapy, chemotherapy alone or in combination, comprising of stage I to stage IV disease, participated in this study. Majority of patients belonged to age group 40-59 years accounting for 58.81%.

Data were collected prospectively using a questionnaire derived directly from the published LENTSOMA scales during a personal interview with the patients. In the questionnaires the subjective questions from the ‘vagina’ and ‘sexual dysfunction-female’ sections were included. In the published scales each answer is scored from 1 to 4 depending on the severity of the symptoms, the higher the score the more severe the symptom. As there are four parts to the published vagina subsection, the maximum subjective score was 16 and similarly for the sexual dysfunction scale scores. The final score was recorded in two ways: as the highest or maximum for vagina and sexual dysfunction scales and as an average score for each scale. If more than 50% of the questions were not answered then the average score was defined as missing. The score was converted to a mean by dividing by the number of questions answered. 

*Statistical analysis*

**Figure 1:**
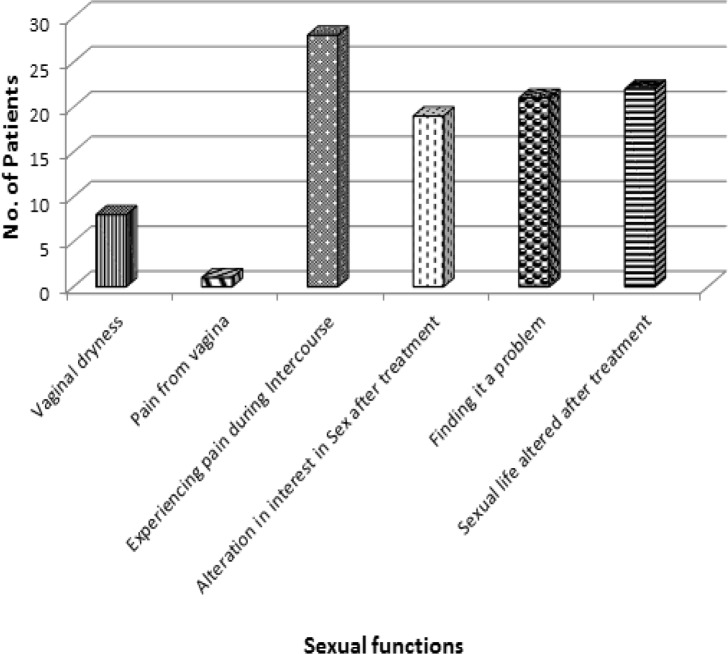
Distribution of Patients on the Basis of Sexual Function and Sexual Satisfaction

Data were entered onto a computer database and analyzed using SPSS (Statistical Package for Social Sciences) version 18.0. The mean late effects on normal tissue subjective vaginal and sexual dysfunction subscale scores were not normally distributed (Figure 1) and so non-parametric tests were used. For the analysis with age, patients were divided into five groups: 20-29 years, 30-39 years, 40-49 years, 50- 59 years, 60- 69 years.

## Results

*Result and Analysis*

An analysis was made for 85 patients who were treated with surgery, radiotherapy, chemotherapy alone or in combination and late effects on normal tissue was recorded in patients who presented in radiation oncology outpatient department. Findings of this study showed that pain during intercourse and altered sexual life were reported by 32.9% and 25.9% patients respectively whereas 24.7% found it problematic and in 22.3% patients, alteration in interest in sex were reported.Vaginal dryness and ulceration was reported in 9.4% and 1.2 % patients respectively. Vaginal stenosis was seen in 75.29% of patients after treatment with decreased frequency of intercourse after treatment was seen in 16.4 % of patients. Figure 1 is showing all the subjective symptoms with number of respondents.

**Figure 2.**
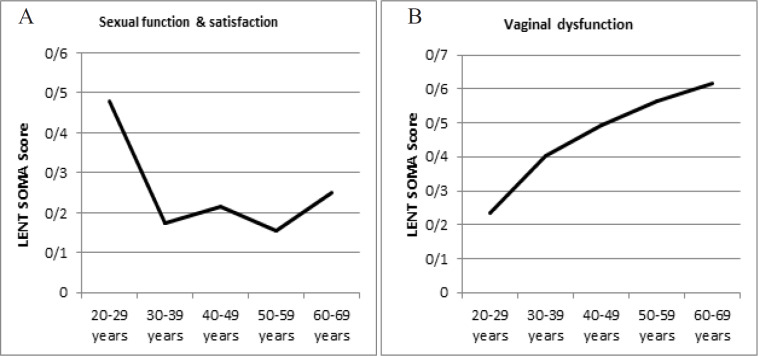
A and B, Age Wise Distribution of Objective LENTSOMA Scores

Mean of the subjective sexual function and satisfaction score was highest in the 20-29 years’ age group (0.4786) (Figure 2 A) and average score was highest in the 50-59 years (0.15) whereas mean score in vaginal dysfunction was highest in the 60-69 years age group (0.6178) (Figure 2 B). 

**Figure 3:**
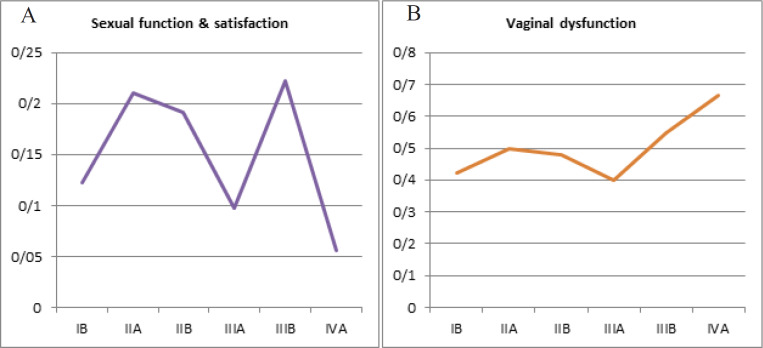
A and B, Stage Wise Distribution of LENTSOMA Scores

Mean of the subjective sexual function and satisfaction scores was highest in stage IIIB disease (0.23) while average subjective sexual score was seen in stage IVA disease (0.06) whereas mean objective score for vaginal dysfunction was highest in stage IVA (Figure 3 A and B).

**Figure 4:**
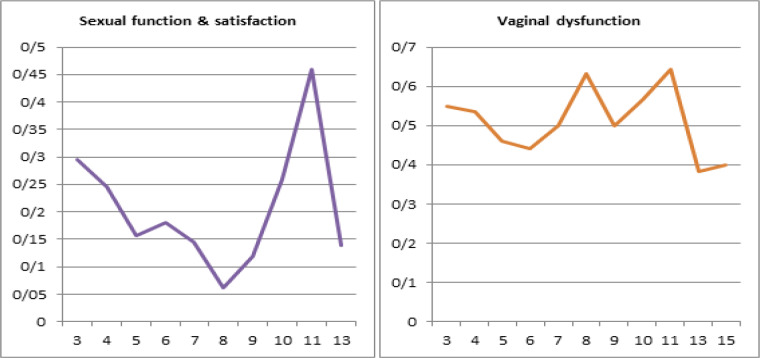
A and B, LENTSOMA Scores According to Gap in Treatment and Interview

Mostly respondents (24.7%) reported that gap between treatment and interview was 4 years followed by 5 years in 12.94% of patients. The mean of subjective sexual function and satisfaction score was highest (0.4601) in patients in which gap between treatment and interview was 11 years. Objective mean score for vaginal dysfunction was highest (0.6444) in patients who were interviewed after 11 years of treatment (Figure 4 A and B).

**Figure 5:**
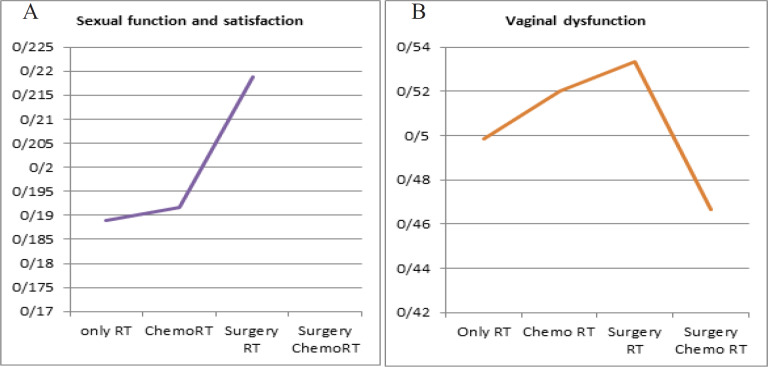
A and B, LENTSOMA Scores in Various Treatment Modalities Used alone or in Combination

The mean subjective sexual function and satisfaction score was highest (0.4601) in patients who were treated in 1999 when compared with other patients (p value-0.093). Mean for sexual function and satisfaction maximum score was highest in Surgery and radiotherapy (0.94) and minimum in RT alone group. (0.54). Objective mean score for vaginal dysfunction was highest in surgery RT group (Figure 5 A and B).

## Discussion

Sexuality is one of the indicators of quality of life; influencing thoughts, feelings, actions, integrations, and thus, mental and physical health. This is multifactorial and has a complex structure, influenced by biological, psychological, socioeconomic, intellectual, religious and sociocultural factors (Lima Correa et al., 2014). Sexual function is one of the important aspects of quality of life in women treated for cervical cancer. Sexuality post gynecological cancer treatment is important to consider as 40% – 100% of gynecological cancer patients will experience sexual complaints after treatment (Pitcher et al., 2018). Radical treatment of cervical cancer in the form of both surgery as well as radiotherapy results in greater impairment of quality of life along with both physical and psychological effects (Hendren et al., 2005; Juraskova et al., 2003). Therefore, morbidity of these radical treatments needs validated assessment and incorporation into routine clinical scenarios to amplify patient benefits especially due to complexity and the sensitivity of the subject involved (Wenzel et al., 2005).

Findings of this study support the research done by Flay and Matthews (1995) on effects of surgery and radiotherapy in patients treated for cervical cancer in which maximum patients reported dyspareunia after treatment. In most of the studies, women report more of sexual problems than orgasmic difficulties like vaginal dryness, dyspareunia, decreased desire to have sex and less satisfactory sexual life (Abitbol and Davenport, 1974; Frumovitz et al., 2005; Greimel et al., 2009; Bergmark et al., 1999) .

Pain during intercourse were reported in 32.9% followed by altered sexual life in 25.9% of patients whereas 24.7% found it problematic with 22.3% patients showed alteration in interest in sex in this study. Vermeer et al. reported that decrease interest in sex since their treatment was experienced in more than half of the patients along few of them reporting loss of libido as a result of relationship duration, age, or sexuality having become less important (Vermeer et al., 2016). 

In this study, vaginal dryness and ulceration was reported in 9.4% and 1.2 % patients respectively whereas vaginal stenosis was seen in 75.29% on examination during follow up. Sexual dysfunction from surgery is mainly due to the shortened vagina, vaginal dryness, decreased libido. However, after radiotherapy, sexual dysfunction is caused by vaginal stenosis which leads to dyspareunia, difficulty in orgasm, a decrease in sexual satisfaction, and changes in body image (Thapa et al., 2018). 

Only one study by Le Borgne et al., (2013) reported overall improvement in sexual and vaginal functioning of 173 cervical survivors followed up over a long period of 15 years. Radiotherapy induced tissue injury leads to inflammatory changes resulting in ischemia and necrosis in pelvic vasculature and nerve sheaths ultimately progressing to complete fibrosis. The rapidly dividing cells of the vaginal epithelium are particularly sensitive to radiation vaginitis; undergo desquamation, adhesion formation, vaginal wall thinning, reduced elasticity and ultimately stenosis (Kollberg et al., 2015). Frumovitz et al., (2005) studied the quality of life and sexual functioning in cervical cancer survivors. Their subject population was younger than 55 years and mainly belonged to early stage - stages IA and IB, treated with either radical surgery and or radiotherapy. They found lower total sexual function scores in patients treated with radiotherapy. One study has found that women who took radiotherapy have decrease in sexual desires compared to women who only had surgical operation (Aydin and Oskay, 2016). 

According to some studies, sexual dysfunction due to radiotherapy is causally different and most patients with radiotherapy have slightly worse sexual outcomes (White et al., 2013) as findings of this study suggests. Sexual dysfunction from radiotherapy is mainly due to vaginal stenosis (75.29%), as Burns et al., (2007) reported in their research, mainly resulting in dyspareunia. Most studies report more frequent and longer lasting dyspareunia in patients treated with radiotherapy compared to surgery (Schover et al. 1989; Park et al 2007). 

Our study is limited by low data collection rates because of sensitivity of sexual issue discussion in Indian scenario. There was no baseline data available as the sexual function was assessed after treatment. Moreover sexual function findings tend to be influenced by patient’s surroundings and own relationships.

In the future, the results of our study emphasize that patients treated by radiotherapy for cervical cancer should be informed about the potential risk of sexual and vaginal problems. Currently it is an underestimated and neglected problem due to time constraints, lack of privacy and the reluctance among patients and health professionals (White and Faithfull, 2013; Flynn et al., 2012; White et al., 2011). Most studies indicate that only about 15% of gynecological cancer patients had been briefed on expected sexual effects during and after treatment (Tangjitgamol et al., 2007; Tsai et al., 2011). 

Currently there is no single self-report measure in routine clinical follow up use which is brief, easy to complete and incorporates all (physical, psychological, emotional) aspects of sexual health for people affected by cancer (Nagele et al., 2015). We used LENTSOMA scale in our study as it provides a comprehensive system to assess treatment induced sexual difficulties to score the impact of radiotherapy morbidity in cervical cancer. It has also highlighted the importance of obtaining pre- radiotherapy scores (Routledge et al., 2003). 

The findings of this study suggested that the combination of surgery and radiotherapy in cervical cancer patients caused more sexual dysfunction and dissatisfaction, especially in lower age group. Treatment morbidity in term of sexual function was more with advanced stage disease and with the patients on longer follow up. With increasing survival, Quality of life has become very important and post-treatment sexual dysfunction has also improved with improved technology for radiation delivery. However, there is a need for wide understanding among doctors to address this issue more efficiently.
